# Management of Palatogingival Groove in Maxillary Lateral Incisor: A Report of a Rare Case With a Brief Review of Literature

**DOI:** 10.7759/cureus.46479

**Published:** 2023-10-04

**Authors:** Irfan Ansari, Sanjay Miglani, Vijay Yadav, Shamimul Hasan

**Affiliations:** 1 Conservative Dentistry and Endodontics, Jamia Millia Islamia, New Delhi, IND; 2 Oral Medicine and Radiology, Faculty of Dentistry, Jamia Millia Islamia, New Delhi, IND

**Keywords:** cone-beam computed tomography, treatment, endoperiodontal lesions, palatogingival groove, developmental anomaly

## Abstract

Several morphological abnormalities may occur during tooth development and may be a predisposing factor for periodontal destruction. Palatogingival groove (PGG) is a developmental deformity that may cause localized periodontitis and endodontic complexities. The groove usually originates as a root indentation in the central fossa of the palatal root of maxillary lateral incisors.

Cone beam computed tomography (CBCT) is an excellent radiographic imaging technique capable of identifying PGGs and provides details about the exact site, extent, and depth characteristics of this deformity. Early diagnosis and management of PGGs are of utmost importance, particularly due to their diagnostic intricacies that can pose both clinical and therapeutic challenges.

This article aims to report a rare case of palatogingival groove associated with an on-and-off discharge from the maxillary left lateral incisor tooth (#22). Nonsurgical endodontic treatment was carried out with #22, and the palatogingival groove was sealed with composite restoration after a deep curettage. Excellent radiographic healing was observed after a six-month follow-up.

## Introduction

Palatogingival grooves (PGGs) are developmental anomalies that typically originate as root indentations in the central fossa of the palatal roots of maxillary incisors. These grooves often extend from the cingulum and progress apically along the root surface, varying in length [1-9]. On rare occasions, they can also be observed on the facial surface of the root [4].

Black, in 1908, was the first to recognize and refer to this developmental deformity as a radicular groove [2,5,8-10]. Subsequently, in 1968, Lee et al. provided the initial description of PGGs, delving into their clinical attributes and possible causes [11].

The maxillary lateral incisor is frequently affected tooth due to its embryological challenge of being positioned between the tooth germs of the central incisor and canine [2,12].

Published literature has documented that the prevalence of PGGs ranged from 0.90% to 44.6%. This disparity in the prevalence rates can be ascribed to variations in diagnostic criteria, research methods, ethnic backgrounds, and geographical locations [7-9,12-16].

The exact cause of PGGs remains obscure. However, the prevailing recommendations for the development of PGGs encompass the following four possibilities: (a) an anomaly in embryonic development, potentially involving the folding of Hertwig's epithelial root sheath, (b) a variant of dens invaginatus, (c) genetic factors, and (d) an attempt to form another root [2,5,6,9-12,17-19].

Conventional radiography serves as a non-invasive radiographic imaging technique for acquiring a two-dimensional depiction of the root canal structure. It is practical and cost-effective, making it the primary choice for such purposes. Nevertheless, these imaging techniques provide less detailed, overlapping images with inherent geometric distortions and noise, potentially affecting the accuracy and analysis of these structures. Cone beam computed tomography (CBCT) provides three-dimensional images with higher resolution and detailed accuracy, thus enabling precise evaluation, diagnosis, treatment planning, and post-treatment follow-up [1,2,5,9,10,20].

PGGs offer an excellent harbor for bacterial plaque and calculus aggregation, thus ensuring the onset and progression of periodontal diseases. The clinical implication of PGGs is linked to the occurrence of localized periodontitis, either with or without pulpal involvement, contingent upon the groove extent, depth, and complexity. Deeper grooves may establish a connection with the pulp chamber, leading to pulpal infection. Nevertheless, the primary link between the pulp and the periodontium with a groove is facilitated by accessory canals running along the groove, thus resulting in combined endodontic-periodontic lesions [2,5,9,11,18,19,21].

In the past, teeth with PGGs were typically extracted due to endodontic-periodontal complications and hopeless prognosis [20]. Over the years, numerous treatment options for PGGs have emerged, namely curettage of the affected tissues, elimination of the groove (saucerization), sealing the groove up to the cementoenamel junction, exclusive endodontic and periodontal interventions, combined endodontic-periodontal procedures, and surgical approaches such as guided tissue regeneration and intentional replantation [22].

This article aims to report a rare case of palatogingival groove associated with an on-and-off discharge from the maxillary left lateral incisor tooth (#22). Nonsurgical endodontic treatment was carried out with #22, and the palatogingival groove was sealed with composite restoration after a deep curettage. Excellent radiographic healing was observed after a six-month follow-up.

## Case presentation

**Figure 1:**
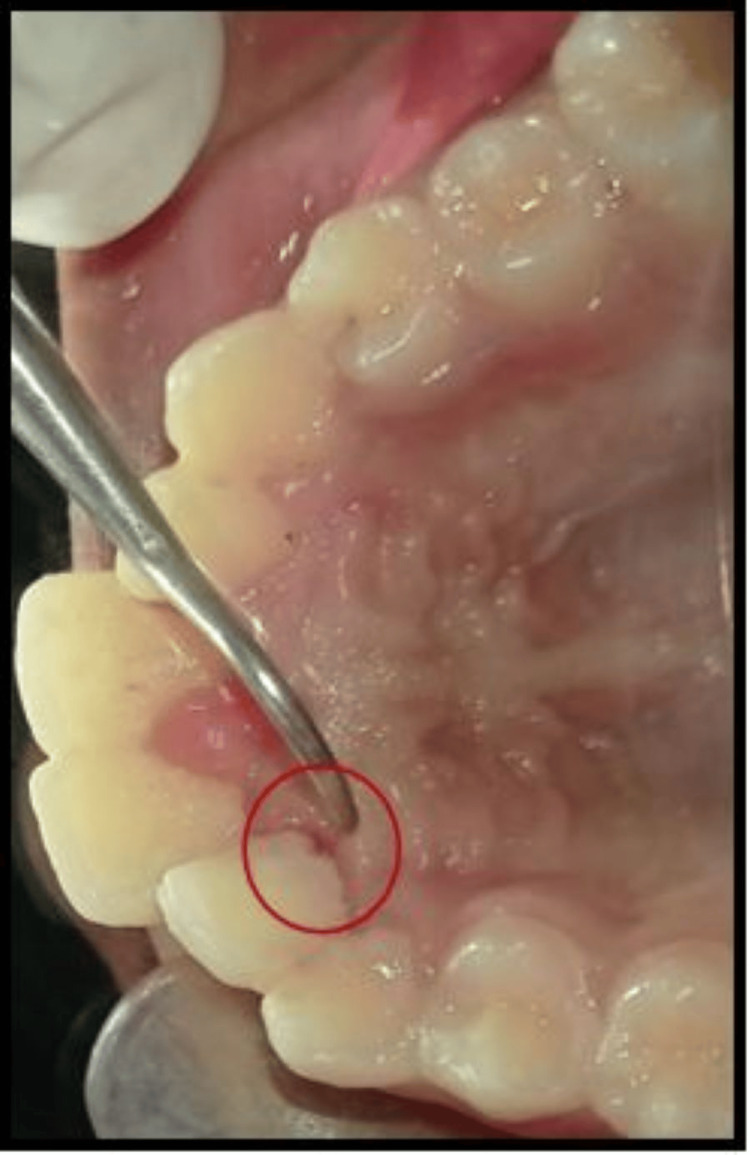
Clinical picture Clinical picture showing a palatogingival groove with #22.

A 16-year-old male patient in good general health reported to our outpatient department with a chief complaint of on-and-off discharge from the palatal aspect of the left maxillary anterior tooth region for the past three months. The patient consulted a private practitioner for the same complaint and was prescribed a course of antibiotics and analgesics for a week (Cap Amox 500 mg thrice daily and Ketorol DT tablet 10 mg twice daily). There was no associated history of trauma. The past medical, dental, and family history were non-significant. No gross facial asymmetry was noticed on extraoral examination. Intraoral examination revealed palatally erupted #12 and #22. The crown of #22 was intact, non-carious, non-tender on percussion, and exhibited a negative response to thermal and electric pulp testing. There were no draining sinus tracts observed on the labial/palatal gingival surfaces. However, a palatogingival groove (PGG) was observed with a mesiopalatal aspect of #22, originating from the cingulum and extending to the gingival sulcus and continuing disto-apically down to the palatal root aspect. Oral hygiene was fair with a mild pocket (4 mm) on the palatal mucosa of #22 (Figure 1).

**Figure 2:**
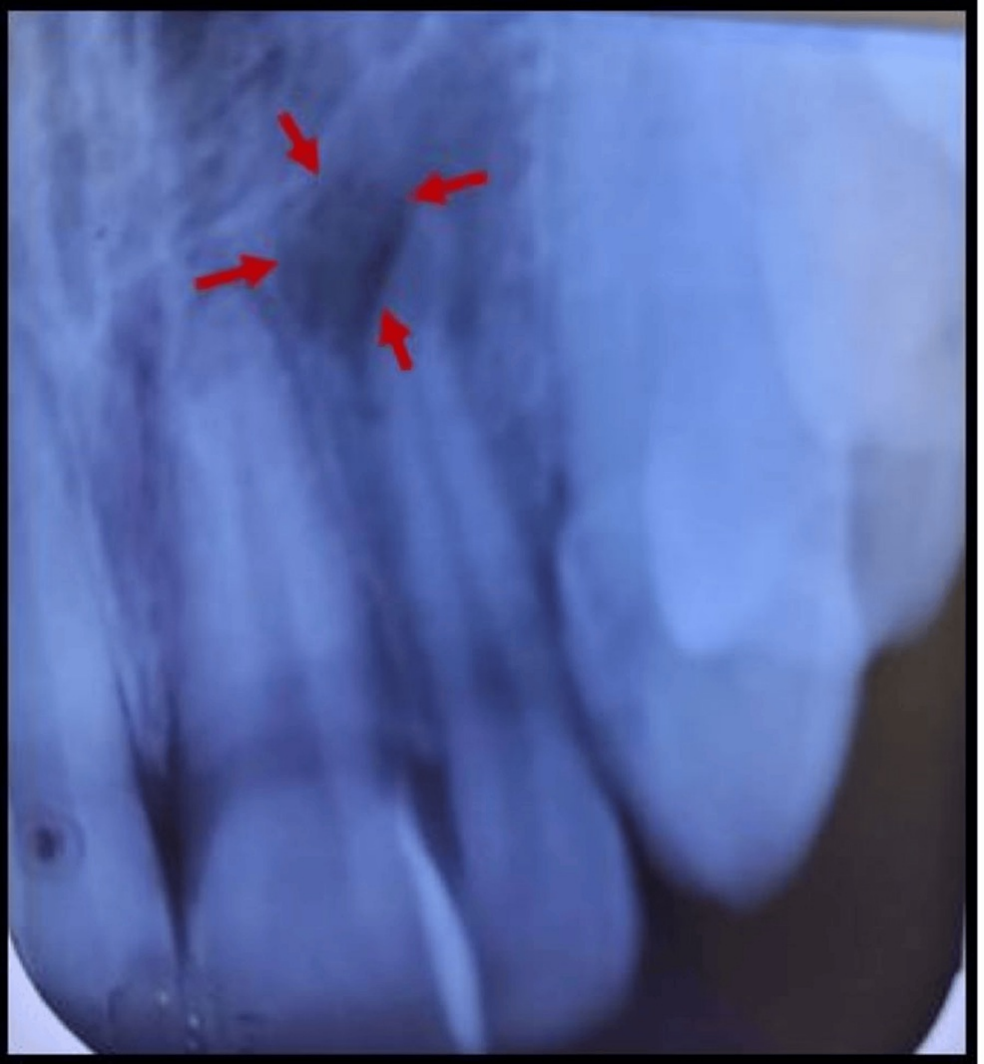
Intraoral periapical radiograph (IOPAR) Intraoral periapical radiograph showing an ill-defined periapical radiolucency with #22.

An intraoral periapical (IOPA) radiograph revealed an intact crown of #22 with an ill-defined, hazy radiolucency in the periapical region and dilacerated apical one-third of the root (Figure 2).

A CBCT scan was advised to evaluate the exact location, depth, and extent of the PGG. CBCT scan with a maxillary field of view was acquired with iCAT model 17-19 imaging system (Imaging Sciences International system, Hatfield, PA) at 120 KV, 5 mA, an exposure time of 7 s, with voxel size of 0.25 mm × 0.25 mm × 0.25 mm and image matrix of 640 × 640.

**Figure 3:**
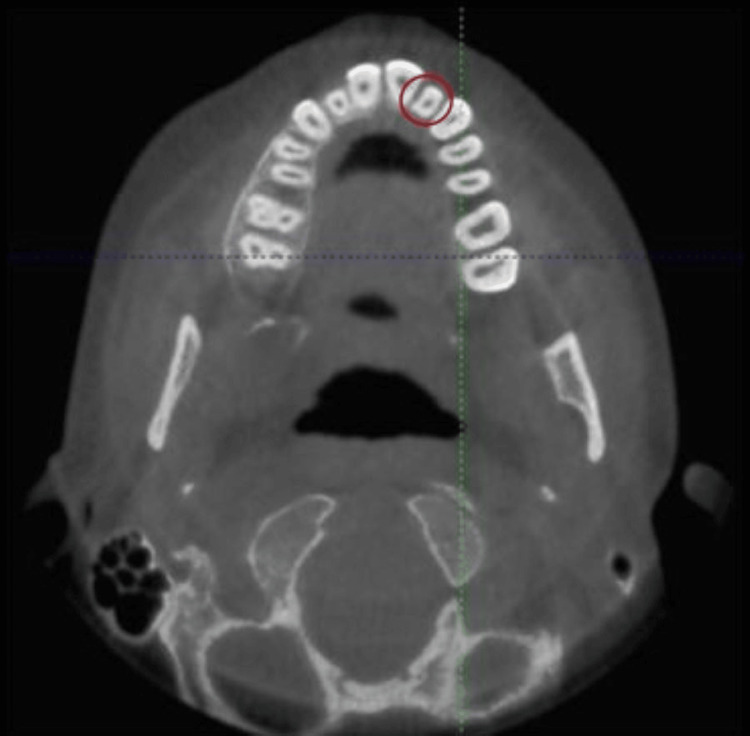
Axial CBCT image Axial CBCT section showing type I palatogingival groove with #22. CBCT: Cone beam computed tomography.

The axial view revealed a shallow palatogingival groove at the level of the cementoenamel junction with a normal root canal configuration (type I groove) (Figure 3).

**Figure 4:**
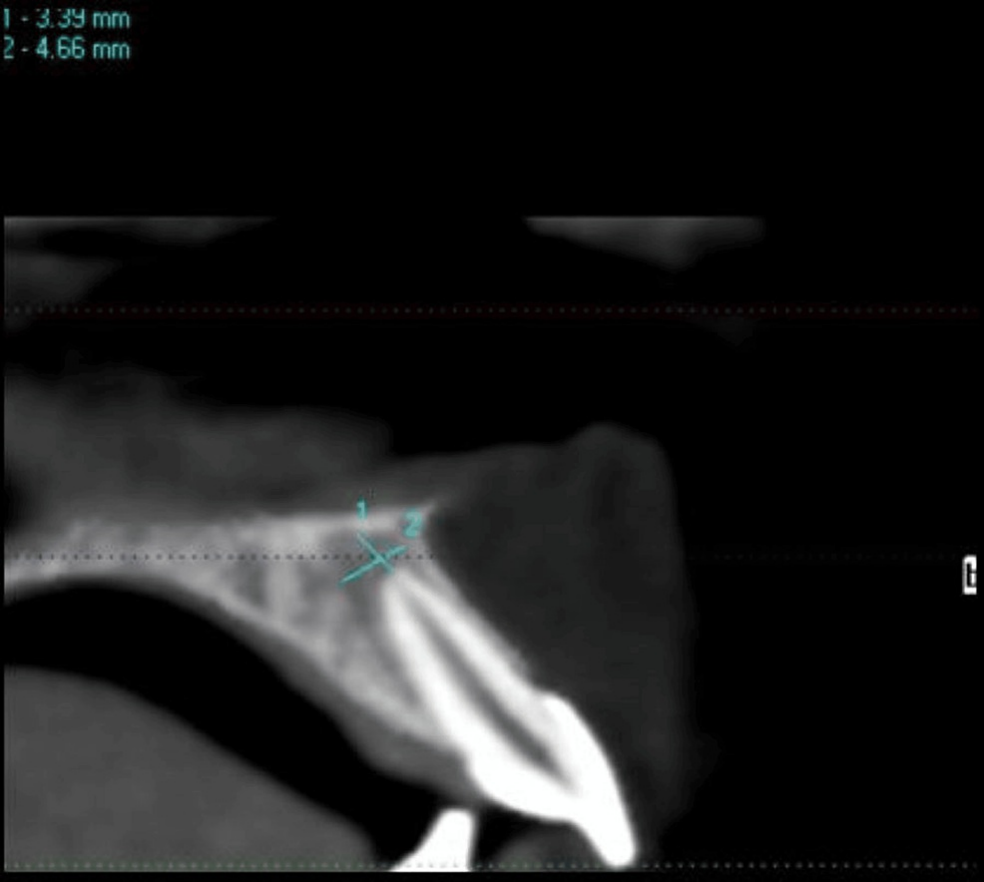
Sagittal CBCT image Sagittal CBCT section showing a hypodense lesion at the periapex of #22. CBCT: Cone beam computed tomography.

The sagittal section revealed an ill-defined (3.39 mm x 4.66 mm) periapical hypodensity with intact cortical plates of #22 (Figure 4).

Taking the clinical-radiographic findings into account, a final diagnosis of chronic periapical abscess secondary to the palatogingival groove with #22 was made.

The treatment plan was explained to the patient and his parents. After a written informed consent, the patient was scheduled for nonsurgical endodontic treatment with #22.

Infiltration anesthesia was given to the patient, and a rubber dam application was done. Access opening was done using a size 2 round bur and an Endo Z bur (Dentsply Sirona, Charlotte, North Carolina). Canal patency was achieved using a #10 K-file followed by coronal flaring with G.G. drill numbers 1, 2, and 3 in a step-back manner. The working length was found to be 21 mm using a radiographic technique and confirmed with an electronic apex locator (Root Zx Mini). Biomechanical preparation was done in a step-back manner up to master apical file #40 with continuous use of 3% sodium hypochlorite as an irrigant and intermittent use of saline and ethylenediaminetetraacetic acid (EDTA). Calcium hydroxide was used as an intracanal medicament, and a closed dressing was given. The patient was recalled after three days, and the canals were irrigated with 3% sodium hypochlorite, saline, and EDTA as per standard protocol. Canals were dried, and a long-term dressing of calcium hydroxide and Iodoform mixture (METAPEX) was placed in the canals. There was no pain on percussion with #22 after a week of follow-up.

**Figure 5:**
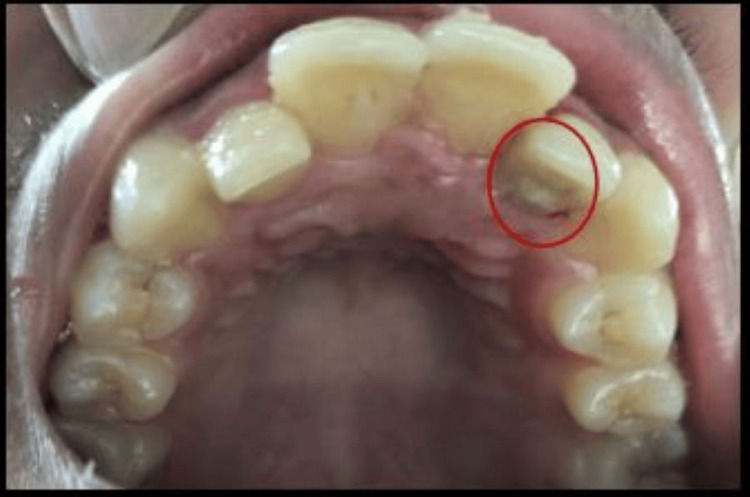
Post-treatment picture Root canal treated #22 and sealed palatogingival groove.

The patient was recalled after one month for obturation of the canal. The obturation of the canal was completed using the lateral condensation technique. Deep curettage of the palatogingival groove was done, and it was sealed with composite restoration (Figure 5).

**Figure 6:**
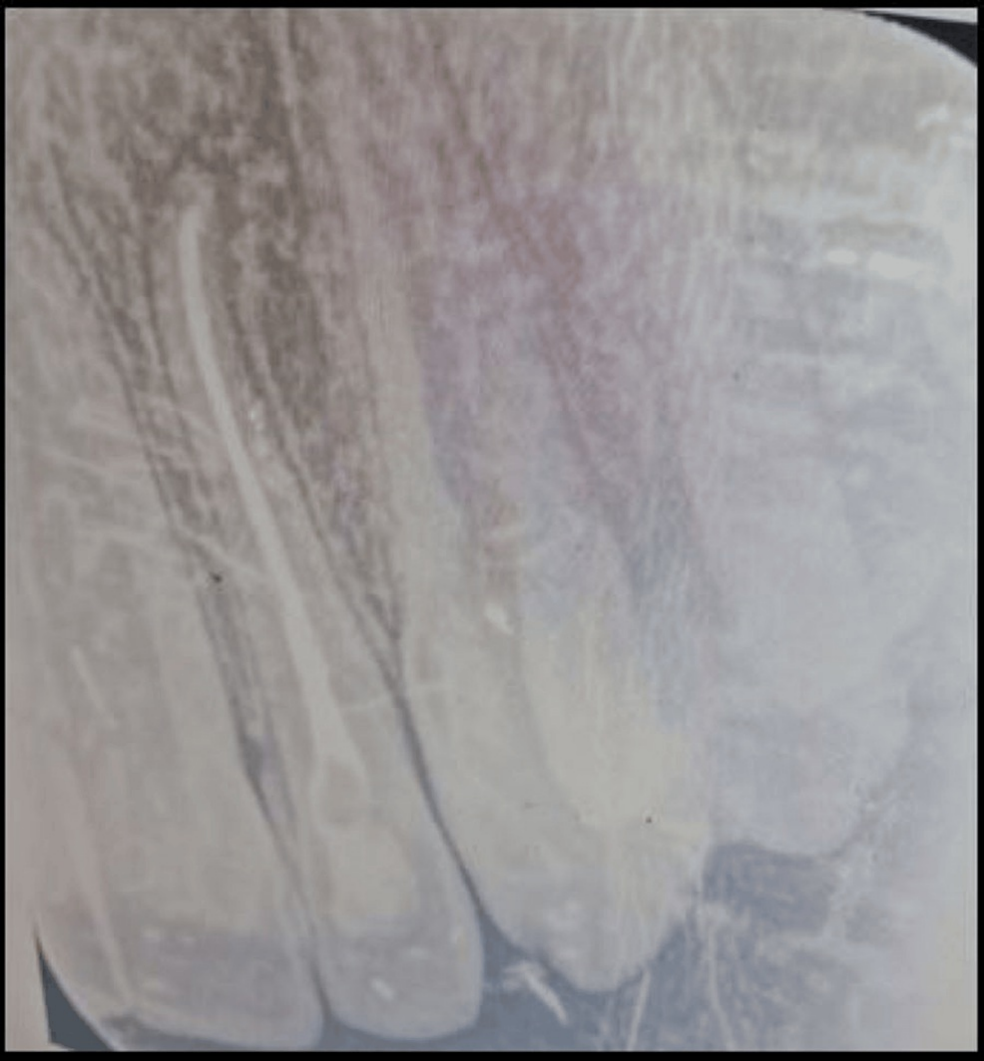
IOPAR at the six-month follow-up IOPAR showing complete healing at the periapical region of #22. IOPAR: Intraoral periapical radiograph.

Follow-up was done every month. Complete radiographic healing was observed periapically with #22 at the six-month follow-up (Figure 6).

## Discussion

The PGG serves as an excellent gateway for oral microorganisms to penetrate periodontal tissues, resulting in periodontal destruction, along with concurrent pulp necrosis and/or apical periodontitis, thus posing a threat to diagnosis and treatment plans [23].

Numerous classification systems have been proposed to classify PGGs due to notable discrepancies observed in their location, extent, depth, and termination [8,14,24,25] (Table 1).

**Table 1:** Classification system for palatogingival groove This table shows various proposed classifications for classifying palatogingival grooves. CEJ: Cementoenamel junction.

S. No.	Basis of classification	Features
1	Groove location [14]	1) Distal
		2) Mesial
		3) Central (or midpalatal)
2	Groove depth and complexity [8,24]	1) Mild: The grooves are gentle depressions of the coronal enamel which terminate at or immediately after crossing the CEJ.
		2) Moderate: The grooves extend some distance apically along the root surface in the form of a shallow or fissured defect.
		3) Complex: The grooves are deeply invaginated defects that involve the entire length of the root or that separate an accessory root from the main root trunk.
3	Degree of invagination of the groove toward the pulp cavity [8]	1) Shallow/flat (<1 mm)
		2) Deep (>1 mm)
		3) Closed tube that forms a tunnel-like channel
4	Degree of severity based on microcomputed tomography studies [25]	1) Type I: Short groove extending apically not beyond the coronal third of the root and with a normal root canal configuration
		2) Type II: Long shallow groove extending beyond the coronal third of the root and with a normal or simple root canal
		3) Type III: Long deep groove extending beyond the coronal third of the root and with a complex root canal system

A thorough clinical assessment reveals a funnel-shaped hollow groove with plaque and calculus accumulation, coupled with bleeding upon probing, pocket formation, and clinical attachment loss [2].

Patients with a PGG pathology may experience various symptoms, including occasional dull pain [26], acute pain [27], tooth mobility [28], pain on percussion [26], pus discharge and sinus tracts [27,29], and swollen gingiva. Typically, the onset of this condition might not be apparent until the development of a sinus tract on the alveolar mucosa, thereby presenting a diagnostic and therapeutic conundrum [20].

Occasionally, a few patients may be completely asymptomatic. In the majority of cases, the patient exhibits a negative history of dental caries, tooth discoloration, and trauma. Depending on the pulpal involvement, the pulp may be vital or non-vital. Advanced lesions with deeper grooves often exhibit no response to electric or thermal pulp testing [29].

Published literature has reported that the presence of PGGs correlates with narrow/deep periodontal pockets and overall poorer periodontal health [2,5,12]. However, most cases with type I grooves exhibited mild to no bone loss, possibly due to the shallow nature of type I grooves.

In the presented case, a shallow pocket measuring 4 mm was observed clinically, whereas there was no evidence of bone loss radiographically. Our case findings were in coherence with previously published reports [13,16].

Radiographically, teardrop-shaped radiolucency may be seen in cases with primary pulpal infection, whereas pear-shaped radiolucency with widened periodontal ligament space may be observed in primary periodontal lesions [2]. In a few cases, a para pulpal line may appear either superimposed onto or running parallel to the root canal. This resemblance to a vertical root fracture could potentially lead to misdiagnosis, highlighting the necessity of advanced radiographic imaging regarding morphological anomalies [2,6,19]. Due to two-dimensional representation, conventional radiography lacks the precision required for determining root canal morphology; location and depth of the groove; site, size, and extension of the bone lesion; and the relationship between adjacent structures [2,10].

Microcomputed tomographic evaluation may provide a three-dimensional representation of morphological anomalies. Gu [25] elucidated the relationship between the groove types and the root canal configuration based on microcomputed tomographic examination. However, this method is rarely employed in clinical practice due to its associated high radiation exposure [25], especially in complex cases involving extremely small structures [20].

Over the last decade, CBCT has emerged as a precise diagnostic tool, offering reconstructed three-dimensional images of teeth and adjacent tissues [19]. Regarding PGGs, CBCT imaging can assist in visualizing the pulp-periodontium connection, evaluating the extent and depth of the groove, and revealing the internal configuration of the root canal system [20].

Based on cross-sectional configurations observed in CBCT images, the morphology of extracted teeth, and preoperative radiography, Tan et al. [20] categorized PGGs into three distinct types: type I, characterized by a superficial groove depth, representing a normal, single, and simple root canal; type II, featuring a medium groove depth, denoting a C-shaped canal system; and type III, showcasing a deep groove depth that nearly divides the tooth's root, concurrently revealing two root canals and a normal-shaped apex. This type corresponds to a labial groove communicating with a palatal groove [19,20].

Tan et al. [20] suggested that the axial CBCT images provide optimal visibility of groove depth, thereby aiding in the classification of embryonic grooves based on various anatomical deformities. Consequently, the axial section offers valuable insights into potential treatment complications.

In the presented case, the axial CBCT section revealed a type I palatogingival groove at the level of cementoenamel junction corresponding to a normal, single root canal. The sagittal CBCT section showed a hypodense lesion at the periapex of #22 with intact cortical plates.

Published literature has utilized diverse methodologies to evaluate the prevalence of PGGs. These methods include microscopic examination and in vitro microcomputed tomography. For in vivo evaluations, clinical examination, radiographic examination, and CBCT were employed. The findings, however, demonstrate variability [2,5,7-9,12-14,16,18,30-34] (Table 2).

**Table 2:** Prevalence of palatogingival grooves This table lists the studies depicting the prevalence of palatogingival grooves on maxillary anterior teeth. PCG: Palatogingival groove; MCIs: Maxillary central incisors; MLIs: Maxillary lateral incisors; CBCT: Cone beam computed tomography.

S. No.	Study	Sample size	Methodology	Prevalence of PGGs			
				Patient with PGG (%)	Teeth with PGG (%)	MCIs with PGG (%)	MLIs with PGG (%)
1.	Everett et al. [13]	625 maxillary lateral incisors	Survey of extracted teeth				2.88
2.	Withers et al. [12]	531 individuals, 1045 maxillary lateral incisors, and 1054 maxillary central incisors	Clinical examination	8.5	2.33	0.28	4.4
3.	Kogon [8]	3168 teeth; 1786 MLIs and 1382 MCIs	Survey of extracted teeth		4.6	3.4	5.6
4.	Bacić et al. [14]	1081 individuals (aged 20–22), 634 individuals with periodontal disease (aged 35–50 years)	Clinical examination	1.01; 0.79			
5.	Hou et al. [16]	Chinese, 101 individuals, 202 MLIs, and 202 MCIs	Clinical examination (clinical probing, radiography, surgical flap operation, and an enlarging dental mirror)	44.6	18.1	5.9	30.2
6.	Pécora et al. [30]	500 MLIs and 421 MCIs	Survey of extracted teeth		2.3	2	2.6
7.	Pécora et al. [15]	642 individuals	Clinical examination (magnifying glass and dental explorer)	3.9		0.9	3
8.	Storrer et al. [31]	2006, 73 MLIs	Survey of extracted teeth				9.58
9.	Kim et al. [2]	2011 Saudi; 276 men (aged 11–55 years)	Clinical examination dental (a mouth mirror and dental explorer)				10.3
10.	Arslan et al. [7]	Turkish; 416 individuals; 651 MLIs and 674 MCIs	CBCT	4.1		0.6	2.3
11.	Zhang et al. [5]	Chinese; 1715 individuals; 3430 MLIs and 3430 MCIs	CBCT	8.40	2.40	0.29	4.5
12.	Al-Rasheed et al. [32]	276 Saudi individuals	Clinical examination	10.3, 6.5%, 3.8 for coronal and apical grooves, respectively			
13.	Aksoy et al. [33]	191 patients (age range: 16-80); 993 maxillary anterior teeth	CBCT	0.5	4.18	0.57	2.22
14.	Lekshmi et al. [18]	119 patients (mean age: 31.6 ± 13.5 years); 636 maxillary anterior teeth	CBCT	1.88		1.47	4.2
15.	Alkahtany et al. [9]	490 patients screened; 264 patients included (age range: 18-80 years)	CBCT				4.9
16.	Varun et al. [34]	163 lateral incisors	CBCT	7.3			7.3

The treatment objectives for a tooth with a PGG are achieving complete elimination of microorganisms, ensuring a thorough and permanent seal of the groove, and achieving complete healing of the periodontium [2]. The recommended treatment options encompass deep curettage of damaged periodontal tissues, saucerization of the groove followed by filling it with restorative material, regenerative procedures, intentional replantation, and extraction [2,10].

Cases with shallow grooves without any periodontal damage may be treated with superficial odontoplasty and the removal of granulation tissue through periodontal procedures. Conversely, when grooves are moderate to complex, a combined approach involving both endodontic and periodontal interventions is required [10].

Various materials have been employed to seal PGGs. Due to their superior properties, mineral trioxide aggregate (MTA) and iRoot BP Plus have emerged as excellent substitutes for previously used materials such as amalgam or glass ionomer [19,20].

In a recent study, Tan et al. [20] advocated that intentional replantation should be re-evaluated as a treatment option for teeth that were previously considered irreparable. The intentional replantation procedure facilitates the clinicians to efficiently observe the extent, depth, and exact site of palatal grooves, thereby allowing for thorough infection debridement with ease.

The prognosis for a tooth with PGG is contingent on multiple factors, such as the groove location, access to the anomaly, accompanying periodontal disease severity, and the characteristics of the groove (shallow/deep or short/long) [2,3,14].

The groove's depth plays a significant role in shaping the configuration of the root canals, potentially posing challenges for achieving effective endodontic sealing. Teeth featuring shallower grooves with less depth extending along the root generally exhibit a more favorable prognosis [20]. Moreover, the prolonged inflammation and transosseous bone defects impede the development of a new periodontal attachment. Therefore, the assessment of the prognosis remains prudent and reserved, despite the excellent results observed during a one-year follow-up [19].

Recommendations

PGG serves as a “plaque trap” resulting in periodontal diseases with or without endodontic implications. Detailed history, clinical evaluation, and CBCT facilitates an early and accurate diagnosis. Treatment options vary and encompass saucerization and sealing the defect, endodontic therapy, and surgical periodontal procedures. Type I grooves are usually treated with saucerization, curettage, and sealing of the groove and may require endodontic treatment where pulpal involvement occurs. Type II and III grooves necessitate complex endodontic and periodontal interventions.

## Conclusions

Our patient presented with a complaint of discharge from the maxillary anterior tooth region. After a detailed history and clinical and radiographic examination, a diagnosis of chronic periapical abscess secondary to a palatogingival groove with #22 was made. Nonsurgical endodontic treatment was carried out with deep curettage and sealing of the groove. A six-month follow-up showed favorable radiographic healing with no recurrence episodes.
